# Physical Properties and Reactivity of Microdomains in Phosphatidylinositol-Containing Supported Lipid Bilayer

**DOI:** 10.3390/membranes11050339

**Published:** 2021-05-03

**Authors:** Toshinori Motegi, Kingo Takiguchi, Yohko Tanaka-Takiguchi, Toshiki Itoh, Ryugo Tero

**Affiliations:** 1 Electronics-Inspired Interdisciplinary Research Institute, Toyohashi University of Technology, Toyohashi 441-8580, Japan; 2 Division of Biological Science, Graduate School of Science, Nagoya University, Nagoya 464-8602, Japan; j46037a@cc.nagoya-u.ac.jp (K.T.); yohko-tk@sd6.so-net.ne.jp (Y.T.-T.); 3 Biosignal Research Center, Kobe University, Kobe 657-8501, Japan; titoh@people.kobe-u.ac.jp; 4 Department of Applied Chemistry and Life Science, Toyohashi University of Technology, Toyohashi 441-8580, Japan

**Keywords:** lipid bilayer membrane, F-BAR protein, phosphatidylinositol, atomic force microscope, single particle tracking

## Abstract

We characterized the size, distribution, and fluidity of microdomains in a lipid bilayer containing phosphatidylinositol (PI) and revealed their roles during the two-dimensional assembly of a membrane deformation protein (FBP17). The morphology of the supported lipid bilayer (SLB) consisting of PI and phosphatidylcholine (PC) on a mica substrate was observed with atomic force microscope (AFM). Single particle tracking (SPT) was performed for the PI+PC-SLB on the mica substrate by using the diagonal illumination setup. The AFM topography showed that PI-derived submicron domains existed in the PI+PC-SLB. The spatiotemporal dependence of the lateral lipid diffusion obtained by SPT showed that the microdomain had lower fluidity than the surrounding region and worked as the obstacles for the lipid diffusion. We observed the two-dimensional assembly of FBP17, which is one of F-BAR family proteins included in endocytosis processes and has the function generating lipid bilayer tubules in vitro. At the initial stage of the FBP17 assembly, the PI-derived microdomain worked as a scaffold for the FBP17 adsorption, and the fluid surrounding region supplied FBP17 to grow the FBP17 domain via the lateral molecular diffusion. This study demonstrated an example clearly revealing the roles of two lipid microregions during the protein reaction on a lipid bilayer.

## 1. Introduction

The lateral diffusion and assembly of lipids and proteins in and on lipid bilayer membranes are fundamental key factors of various reactions in plasma membranes, such as transportation of materials and signals into and out of cells. Spatiotemporal scales of the assembled structures, varying from nm to µm, e.g., clusters and domains, are proposed depending on the reaction [1,2]. It has been proposed that domains with submicron sizes play crucial roles [1,2]. However, the relation between the properties and functions of the assembled structures of lipid bilayer membranes during the reactions with proteins are still elusive. Colocalization of specific lipids and proteins, e.g., sphingomyelin, cholesterol, and transporter proteins has been proved in previous studies, but the relation between the properties of the lipid domains, and the mechanism and roles of the colocalization are not always clear. 

In the previous studies of lipid localization, phase separation and domain formation in lipid bilayer membranes, intermolecular interaction at the hydrophobic core have been investigated in detail, for example, the phase transition of acyl chains between liquid crystalline and gel phases, and higher affinity of cholesterol to saturated acyl chains than to unsaturated ones [3]. On the other hand, localization of lipids with large hydrophilic head group, such as phosphatidylinositol (PI) and glycolipids, is less understood, probably because their large hydrophilic head groups cause competition between the attraction via hydrogen bond and the hydration (fluctuation) repulsion [4]. Redfern and Gericke showed that PI-enriched microdomains are formed in lipid vesicles consisting of PI and phosphatidylcholine (PC) at pH > 6.5 even at the temperature higher than the phase transition temperatures of the PI and PC [5]. Recent theoretical study also shows the clustering of PI derivatives in PC membranes [6]. They proposed fluid-fluid demixing induced by the hydrogen bond between the inositol rings of PI. PI and their phosphorylated derivatives are lipid mediators in many membrane trafficking events in eukaryotic cells [7,8,9,10]. It was proposed that the generation and the local accumulation of these specific lipids and proteins are precisely controlled at the moment of during the signaling reaction such as allergic reactions [11,12]. 

Structural transformation of cell membranes is an important process of membrane traffic, and is induced by assembly of membrane deformation proteins, e.g., caveolin, clathrin, and BAR proteins, which activities are strongly related to PI derivatives [13,14,15,16,17,18]. Tanaka-Takiguchi et al. reported that purified F-BAR proteins retain the function of tubular growth from giant unilamellar vesicles (GUVs) consisting of PI and PC, and that their tubulation activities are classified to two types; FBP17 and Cip4 developed many protrusions simultaneously over the whole GUV surface, while PSTPIP1 and pacsin-2 developed a few protrusions [19]. The results indicate that each of the F-BAR proteins behaves differently at the initial processes of assembly, e.g., adsorption, diffusion, and nucleation, on the lipid bilayer containing PI-enriched domains. 

Here, we investigated the morphology, physical property, and reactivity of microdomains in a supported lipid bilayer (SLB) consisting of PI and PC, by the atomic force microscope (AFM) observation and single particle tracking (SPT) measurement. SLB is an artificial lipid bilayer system formed at solid-liquid interfaces. It is a useful biomembrane model system to investigate activities of membrane proteins in and on lipid bilayer [20,21,22,23,24,25]. Recently, there have been studies on the construction of SLB systems containing specific lipid domains to investigate the function of peptides acting specifically on the domains [26,27,28]. We aimed to characterize the properties of the microdomains, and to clear their roles at the initial process of the protein assembly reaction on the lipid bilayer.

## 2. Materials and Methods

### 2.1. Supported Lipid Bilayer

The SLBs comprising PI (Liver, Bovine) and PC (Egg, Chicken) at the molar ratio of 1:1, which is the component showing the tubular growth in the previous study [19], were prepared on a mica substrate by the vesicle fusion method [29,30]. One of two fluorescent probes, which show different distribution in the supported lipid bilayer, 4,4-difluoro-5,7-diphenyl-4-bora-3a,4a-diaza-s-indacene-3-dodecanoic acid (β-BODIPY® 530/550 C5-HPC, Molecular Probes, Eugene, OR, USA) (BODIPY-HPC) and 1, 1′-Dioctadecyl-3, 3, 3′, 3′-tetramethylindocarbocyanine perchlorate (Molecular Probes) (DiI-C18) was mixed with PI and PC at the molar ratio of 0.2% for the epi-fluorescence microscopy, and 1 × 10^−6^% for SPT. The chloroform solution of the mixed lipids was dried in a glass vial with N_2_ stream and evacuated for >6 h. After addition of a buffer solution (100 mM KCl, 25 mM HEPES/ NaOH pH 7.4) into the dried lipid film, the unilamellar vesicle suspension was prepared through the processes of agitation, freeze-thaw cycles, and extrusion through a 100 nm-pore polycarbonate filter. The supported lipid bilayer was formed through the immersion of a freshly cleaved mica substrate into the vesicle suspension and the incubation at 45 °C for 60 min following the previous protocols [30,31,32]. After the incubation, the excess vesicles were washed out by exchanging the vesicle suspension with the buffer solution repeatedly. We kept the PI+PC-SLB sample in the buffer solution during the sample preparation, epi-fluorescence microscope observation, AFM observation, and SPT measurement. 

In this study, the F-BAR domain region of FBP17, which has the same activity as the whole length FBP17, was used as FBP17. FBP17 was expressed and purified following procedures in the previous report [19]. Briefly, cDNA encoding human FBP17 was obtained by PCR using primer sets designed according to database sequences, and the cDNA fragment was subcloned into pGEX6P-1 (GE-Healthcare, Little Chalfont, UK). The partial construct of GST-FBP17 F-BAR domain (amino acids 1−300) was PCR amplified and subcloned into expression vector. The recombinant FBP17 was obtained from a bacterial expression system, and the GST tag was removed by means of on-bead cleavage with PreScission proteases (GE Healthcare, Little Chalfont, UK). The released protein was dialyzed in a buffer solution (100 mM NaCl, 25 mM HEPES, 5 mM EDTA/ NaOH pH 7.4). The diluted protein solution was added to the sample cell containing PI+PC-SLB for the AFM observation. 

### 2.2. Apparatus

For the macroscopic observation, an epi-fluorescence microscope (IX51W, Olympus, Inc., Tokyo, Japan) equipped with 60× water immersion objective (N.A. 1.00) was used. The spatial resolution at a wavelength of 560 nm was approximately 350 nm based on the Rayleigh criterion. For SPT, an inverted fluorescence microscope (IX-71, Olympus, Inc.) equipped with 100× oil immersion objective (N.A. 1.45) was used. With the light excitation by 532 nm laser, the obtained diffusion of fluorescent probes in the bilayer was recorded by EM-CCD camera (iXon DU-897, Andor Technology, Ltd., Belfast, UK) at the frame rate of 30 frames/s (fps) or 500 fps. The effective pixel size of the SPT recording was 275.86 nm. We observed the surface structure of the PI+PC-SLBs by AFM (5500 Scanning Probe Microscope, Agilent Technologies, Inc., Santa Clara, CA, USA) with magnetic AC mode before and after addition of FBP-17. For AFM imaging, TYPE VI MAC Lever (Agilent Technologies, Inc.) with typical spring constant of 0.2 N/m was used. The epi-fluorescence microscope and AFM observations were performed at an ambient temperature of 23 °C.

### 2.3. Single Particle Tracking 

To perform SPT on a mica substrate, we adopted the diagonal illumination setup (Figure S1 in Supporting Information) [31,32], instead of the conventional total-internal reflection (TIR) condition. We settled the mica substrate with SLB upside-down above the glass coverslip, and then introduced the excitation laser light at a smaller incident angle than that for the TIR condition. Using this setup, we can perform SPT on mica or other materials, without the restriction of optical properties of substrates such as transparency and refractive index [31,32]. The sample temperature was controlled to be 20 °C. In this study, the acquisition rate of 500 fps was achieved at maximum, in addition to the conventional video-rate observation at 30 fps. The position accuracy of the trajectory was calculated to be 44 nm [33]. The time resolution and the location accuracy in this study made it possible to detect the diffusion behavior of lipid molecules in submicron regions. 

The coordinates of the trajectories of the fluorescent probes in PI+PC-SLB were obtained from the movies using an auto-tracking software Particle Tracker [33], which is a plug-in of ImageJ (NIH, USA, https://imagej.nih.gov/ij/). The mean square displacement (MSD) at the time interval of *τ* = *n*Δ*t* was calculated [34,35] for the trajectories continuously tracked for longer than 50 frames and 30 frames, for those obtained at 500 fps and 30 fps, respectively, using Equation (1):

(1) $$MSD=<r{\left(n\Delta t\right)}^{2}>=\frac{\sum_{i=0}^{N-n-1}{\left\{\vec{r}\left(i\Delta t+n\Delta t\right)-\vec{r}\left(i\Delta t\right)\right\}}^{2}}{\sum_{i=0}^{N-n-1}\;},$$

where *N* is the total frame number recorded in a movie, Δ*t* is the time resolution of the movie, and $\vec{r}\left(t\right)$ is the position vector of the fluorescent probe at time *t*. The average MSD over the collected trajectories was calculated as the weighted average on the frame number of each trajectory using Equation (2) [31],

(2) $$<MSD>=\frac{\sum_{j}^{k-1}\sum_{i=0}^{N_{j}-n-1}{\left\{\vec{r}\left(i\Delta t+n\Delta t\right)-\vec{r}\left(i\Delta t\right)\right\}}^{2}}{\sum_{j}^{k-1}\sum_{i=0}^{N_{j}-n-1}\;},$$

where *k* is the number of movies over which the average is calculated, and *N_j_* is the total frame number of the movie *j*. Diffusion coefficient at *τ* (*D*(*τ*)) was obtained from the linear fitting of the average MSD-*τ* plot to <MSD> = 4*D*(*τ*)*τ* at the range from *τ* = 1∆*t* to *τ* = *n*∆*t* [31]. 

## 3. Results and Discussion

### 3.1. Characterization of the Supported Lipid Bilayer Structure

**Figure 1:**
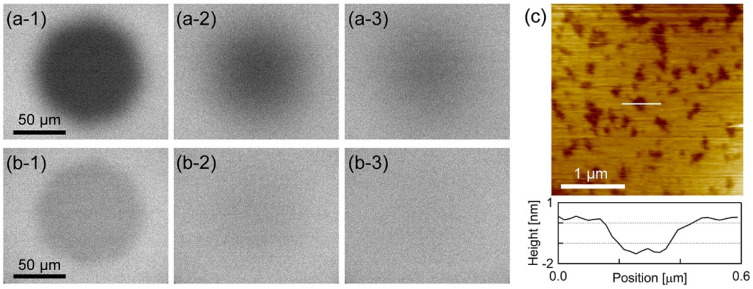
(**a**,**b**) Epi-fluorescence microscope images during FRAP of PI+PC-SLB including (**a**) BODIPY-HPC and (**b**) DiI-C18 obtained at (a-1, b-1) 0 s, (a-2, b-2) 10 min and (a-3, b-3) 20 min after photobleaching. (**c**) AFM topography of PI+PC-SLB and the cross-section profile at the white line.

Figure 1a,b show the epi-fluorescence microscope images during the fluorescence recovery after photobleaching (FRAP) [36,37] process of PI+PC-SLB including BODIPY-HPC and DiI-C18, respectively. Homogeneous fluorescence intensity was obtained over almost the entire sample surface, and the fluorescence intensity recovered in the FRAP observation. The results in Figure 1a,b show that uniform, fluid and continuous PI+PC-SLB was formed on the mica substrate. Figure 1c shows the AFM topography (3.0 × 3.0 µm^2^) of the PI+PC-SLB on the mica substrate. Depression regions of approximately 1.4 ± 0.3 nm (N = 86) depth existed in the PI+PC-SLB. The depression regions were not holes in the SLB but attributed to domains in the lipid bilayer membrane with the different thickness from the surrounding region. Typical thickness of lipid bilayers in AFM topographies are 4−5 nm, and the height difference between lipid domains in SLB is observed as approximately 0.5–1.5 nm [38,39,40]. The lateral size of the depression domains was on the order of 10 nm to 100 nm. The averaged diameter was calculated as 101 ± 7.8 nm (N = 1589 from 9 images). The area fraction of the depression domains was 24 ± 2.2% (N = 9). 

PC-SLB without PI on mica is homogeneous as shown in the previous study [32], because the egg-derived PC is in the liquid crystalline phase at 23 °C. Therefor the depression domains are attributed to a results of the addition of PI. The PI derived from bovine liver has at least one unsaturated acyl chain in its hydrophobic tails and thus is also in the fluid phase; more than half of the acyl chains of the liver-derived PI is unsaturated [41], and the saturated acyl chains are located in the *sn-1* position [42] as with nature-derived PC. PI forms micro domains or clusters in the PI+PC vesicle system via the intermolecular hydrogen bond between PI molecules [5]. The demixing occurs at the temperature higher than the phase transition temperature of PI and PC [5], hence it is not driven by the phase transition at the hydrophobic part of the lipid bilayer, e.g., the phase separation between the gel and liquid crystalline phases. We attribute the depression microdomains in the AFM topography (Figure 1c) to the PI-derived domains. 

It may seem strange that the PI-enriched domains were observed lower than the PC-rich region containing less PI, even though PI has bulky inositol ring on its hydrophilic head group. The AFM topography is, however, a height profile of the constant force between the sample surface and the cantilever tip. In aqueous solutions, it may not simply trace the shape of the sample surface in the presence of additional hydrophilic and hydrophobic interactions [43,44,45]. A representative example is the repulsive hydration force induced by the thermal fluctuation of hydrated substances [4] such as polyethylene glycol (PEG) and glycochains. The hydration force strongly depends on the density and mobility of the hydrophilic substance and the applied force [46]. Recently, Kakimoto et al. showed that the domain comprising PEG-labeled lipid appears lower than the surrounding region that is fluid and contains less PEG-labeled lipid in the amplitude-modulation AFM topography, which was also adopted in this study, although the domain is higher in the frequency-modulation AFM image obtained at zero-applied force [47]. It resembles to the apparent topography in the current study (Figure 1c). Fluctuation of the hydrophilic inositol group on the PI headgroup is suppressed in the PI-derived microdomain via the intermolecular hydrogen bond, whereas the PI in the fluid surrounding region diffuses fluctuating its headgroup. 

The AFM topography in Figure 1c revealed the PI+PC-SLB was heterogeneous microscopically, although the PI+PC-SLB had homogeneous fluorescence intensity in the fluorescence image (Figure 1a,b). The PI-derived domains were not resolved in the conventional optical microscopic observation, because they were smaller than the optical resolution limit. Macroscopic FRAP results in Figure 1a,b indicate that at least the surrounding region of the domains in PI+PC-SLB retained fluidity. We investigated the microscopic fluidity of the two regions in the PI+PC-SLB, the microdomains and their surrounding region, by the SPT method. 

### 3.2. Single Particle Tracking on the Supported Lipid Bilayer

**Figure 2:**
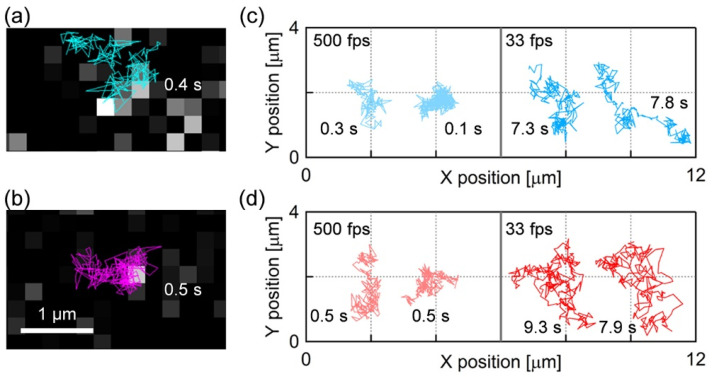
(**a**,**b**) Single molecule fluorescence images and diffusion trajectories of (**a**) BODIPY-HPC and (**b**) DiI-C18 in PI+PC-SLB obtained by SPT at the frame rate of 500 fps. (**c**,**d**) Single molecule trajectories of (**c**) BODIPY-HPC and (**d**) DiI-C18 in PI+PC-SLB. Left and right columns correspond to the trajectories obtained at 500 fps and 33 fps, respectively. The length (measurement time) of each trajectory is appended.

Figure 2a,b show the single molecule fluorescence images and diffusion trajectories of the fluorescence probes BODIPY-HPC and DiI-C18 in the PI+PC-SLBs, respectively (the movies are shown as Videos S1 and S2 of Supporting Information). Each bright spot in Figure 2a,b was assigned to a single fluorescence probe because of the characteristic properties of the single molecule fluorescence image: homogeneous fluorescence intensity over the bright spots in a sample, single step photobleaching, and density dependence on the fluorescence probe ratio to PI and PC. Figure 2c,d show the typical trajectories of BODIPY-HPC and DiI-C18 molecules in PI+PC-SLB, respectively, obtained at the frame rates of 500 fps and 33 fps. The diffusion of both fluorescence probe was tracked for sufficiently long time for MSD analysis, which we defined ≥ 100 ms (50 frames) at 500 fps and ≥1 s (30 frames) at 30 fps. 

**Figure 3:**
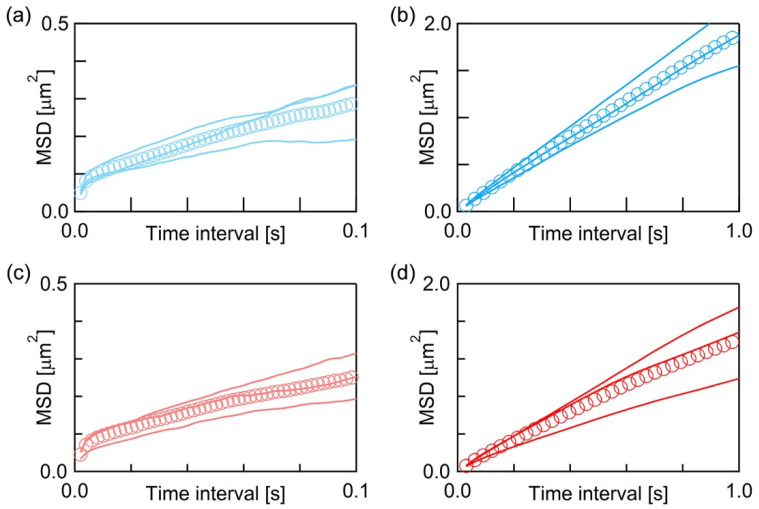
Average MSD-*τ* plots of (**a**,**b**) BODIPY-HPC and (**c**,**d**) DiI-C18 in PI+PC-SLB. The frame rates are 500 fps in (**a**,**c**), and 30 fps in (**b**,**d**).

We obtained more than 10^3^ trajectories at each acquisition rate for both fluorescence probes and calculated their average MSD (Equations (1) and (2)). Figure 3 shows the average MSD-*τ* plots of BODIPY-HPC at 500 fps (Figure 3a) and at 30 fps (Figure 3b), and those of DiI-C18 at 500 fps (Figure 3c) and at 30 fps (Figure 3d). In each of Figure 3a–d, three solid curves are the average MSD-*τ* plots obtained from three independently prepared PI+PC-SLB samples, and their overall average is plotted as markers. MSD increased with *τ*, but not linearly in all the average MSD-*τ* plots. Random diffusion, which is called normal diffusion, gives linear dependence between MSD and *τ*, while the diffusion in a heterogeneous medium shows non-linear MSD−*τ* dependence [35,48,49]. This phenomenon is categorized as anomalous diffusion, and the analysis of the spatiotemporal dependence of the diffusion behavior provides the information of inner structures and their physical properties in a heterogeneous medium. As shown in Figure 3, the average MSD−*τ* plots showed obvious non-linearity, thus we further investigated spatiotemporal dependence of the molecular diffusion in detail. 

**Figure 4:**
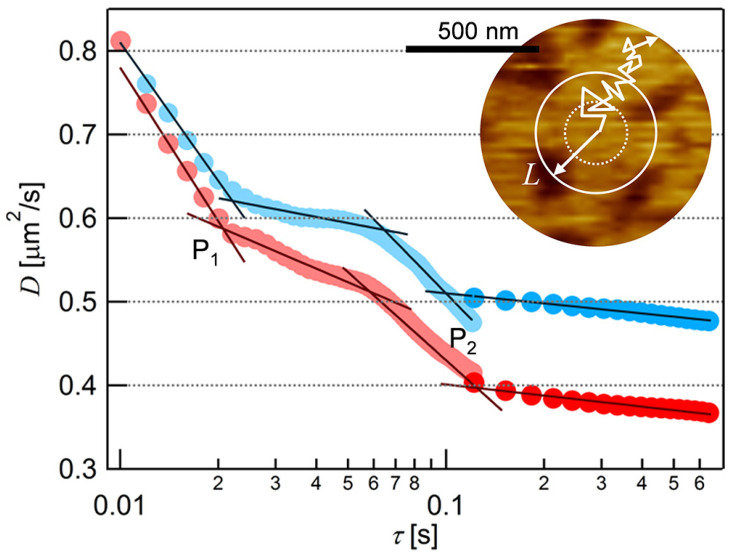
The dependence of diffusion coefficient (*D*) on time interval (*τ*) obtained from the analyses of the average MSD-*τ* plots of BODIPY-HPC (the blue plot) and DiI-C18 (the red plot) in PI+PC-SLB. The two infection points, P_1_ = (*τ*_1_, *D*_1_) and P_2_ = (*τ*_2_, *D*_2_), were obtained as the intersection of the extrapolated fitting lines (black lines) at the downward and flat regions of the *D*-*τ* plot. Inset: Schematic illustration of the diffusion behavior of a fluorescent probe superimposed on the AFM topography of the PI+PC-SLB.

We obtained *D* at each *τ* from the average MSD-*τ* plots in Figure 3 and plotted them in Figure 4. In both cases of the two fluorescence probes, the value of *D* decreased with *τ* in the range of *τ* < 0.02, and 0.06 < *τ* < 0.1, and finally approached to constant at *τ* > 0.1. Two infection points, at which a downward slope curve turns to flat, were present in each plot at *τ* ~ 0.02 s and *τ* ~ 0.1 s (P_1_ and P_2_ in Figure 4). This is a typical behavior of the anomalous diffusion caused by the diffusion obstacles [48,49]. In the case of the PC-SLB without PI on mica, *D* did not depend on *τ* (Figure S2 in Supporting Information) because the PC-SLB is two-dimensionally homogeneous as mentioned above [32]. The trajectories obtained at 30 fps (Figure 2) show that the diffusion distances extended to several micrometers, therefore indicate the fluid region was continuous in this spatial range. We interpret the AFM topography in Figure 1c as that the microdomains worked as diffusion obstacles and that the surrounding region was fluid. 

This characterization in fluidity is reasonable from the viewpoint of the diffusion length estimated from of the *D*-*τ* plot (Figure 4). The two infection points in the *D*-*τ* plot of BODIPY-HPC (the blue plot in Figure 4) were P_1_ = (*τ*_1_, *D*_1_) = (0.022, 0.62) and P_2_ = (*τ*_2_, *D*_2_) = (0.10, 0.51), as we obtained from the fitting lines at the downward and flat regions shown in Figure 4 (black lines). More than one infection points are derived from the influences between obstacles and the probe molecule at different spatial scales. There exist two different distributions of sizes in the demixing media composed of the fluid region, where the molecules can freely diffuse, and the obstacles. We deduced the size of the fluid region from the average diffusion distance (*L*) at the infection points using Equation (3) [50]:

(3) $$L^{2}=4D\tau .$$

with substitution of (*τ*_1_, *D*_1_) and (*τ*_2_, *D*_2_) above, *L* were 234 nm and 452 nm, respectively, for BODIPY-PC in PI+PC−SLB. In the case of DiI-C18 (Figure 4, red plot), *L* of 221 nm and 444 nm were calculated from (*τ*_1_, *D*_1_) = (0.021, 0.59) and (*τ*_2_, *D*_2_) = (0.12, 0.40), respectively. We evaluated the domain-free size for diffusion in the surrounding region, by quantifying the density and size of the domains from the AFM topography. Assuming periodic allocation of the circular domain, we estimated the size of the domain-free region to be 261 nm (details are in Supporting Information). It is in good agreement with *L* at the infection point at (*τ*_1_, *D*_1_) above. Therefore, we conclude that the PI-derived microdomains observed in the AFM topography (Figure 1c) were the diffusion obstacles, and thus more rigid than the fluid surrounding region. 

This assignment also supports the interpretation of heights in the AFM topography discussed in the previous section. If we simply assume the same occupying area between PI and PC, the composition of the surrounding region is estimated to be PI:PC ~ 1:2, based on the area fraction of the PI-derived microdomain (24%) and the molar ratio between PI and PC (50:50). The surrounding region is rich in PC but contains sufficient amount of PI. The PI molecules in the surrounding region laterally diffuse fluctuating their headgroup, while the fluctuation of the headgroup is restricted in the microdomain due to the intermolecular hydrogen bond. Additionally, DiI-C18 in PI+PC-SLB (red plot in Figure 4) shows a larger spatiotemporal dependence than that of BODIPY-HPC (blue plot in Figure 4). The larger dependence indicates the stronger affinity between the DiI-C18 with the obstacle. DiI-C18 is a cationic molecule, and the PI head group is negatively charged compared to the PC head group at pH 7.4 [51]. The electrostatic interaction enhanced the obstacle effect of the PI-derived microdomain for DiI-C18.

### 3.3. Roles of Lipid Domains in the F-BAR Protein Assembly

**Figure 5:**
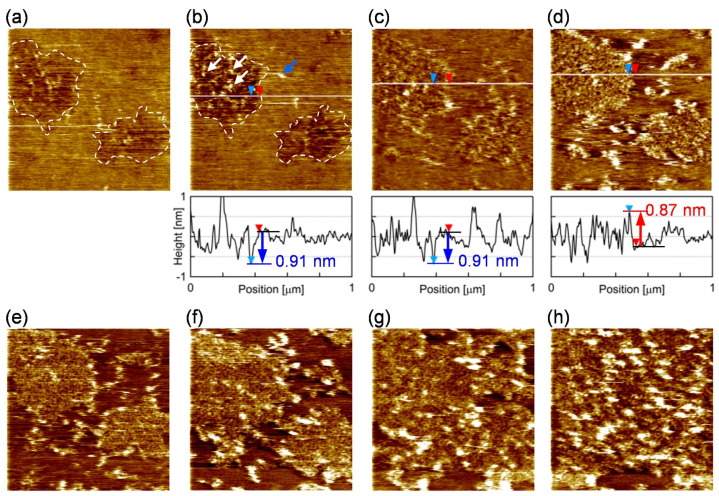
Snapshots from the sequence of AFM topographies (1.0 × 1.0 μm^2^) of the PI+PC-SLB (**a**) 203 s, (**b**) 376 s, (**c**) 897 s, (**d**) 1418 s, (**e**) 1962 s, (**f**) 2679 s, (**g**) 3753 s, and (**h**) 6260 s after the addition of FBP-17. The cross-section profile at the white line is accompanied in (**b**–**d**). The depression microdomains in the view filed is marked with the dashed line in (**a**,**b**). Representative protrusions in the microdomain and the surrounding region were indicated by white and blue arrows, respectively, in (**b**). In the cross-section profiles, the level of the surrounding region is represented by a black line with a red triangle; the level of the microdomain is represented with a blue line or a red line with a blue triangle, in case the microdomain is depressed or protruded from the surrounding region, respectively.

We investigated how the PI-derived microdomain and the PC-rich surrounding region work during the assembly of FBP17 on the bilayer membrane. Figure 5 shows snapshots from the sequential AFM topographies of the PI+PC-SLB after the addition of FBP17 at the concentration of 0.28 µM (the series of AFM topographies are shown as Video S3 of Supporting Information). Two microdomains existed in the view field (indicated by dashed lines in Figure 5a,b). They were observed as depression as with Figure 1c, and additional protrusions appeared after the addition of FBP17 solution (arrows in Figure 5b). The protrusions appeared preferably in the microdomains (white arrows in Figure 5b) and accumulated with time (Figure 5b,c). The height of the protrusions was approximately ~0.9 nm in average, which is in good agreement with the diameter of the F-BAR region of FBP17 [52]. We attribute the protrusions to FBP17 molecules adsorbing on SLB. When the microdomains were filled with FBP17, the domains protruded ~1 nm from the surrounding region (Figure 5d), then the protruded domain grew to the surrounding region (Figure 5e–h). We also found some protrusions in the surrounding region of the microdomains (blue arrow in Figure 5b), but they disappear in the next image. It means that the FBP17 molecules adsorbing on the surrounding region were diffusing. 

These AFM images revealed the roles of the two lipid microregions in PI+PC-SLB at the initial stage of the assembling reaction of FBP17. The rigid PI-derived microdomains worked as scaffolds where FBP17 preferentially adsorbed (Figure 5b,c). The BAR domain proteins including FBP17 are positively charged on their concave face [17], and thus adsorbed to the surface of lipid bilayers containing negatively charged lipids [19]. The fluid surrounding region, in which PI molecules attached with FBP17 laterally diffuse, provided FBP17 to the PI-rich microdomains via the lateral diffusion (Figure 5d–h). The previous study using PI+PC-GUV showed that lipid bilayer membranes are transformed to tubules in the presence of F-BAR proteins including FBP17, whereas PC-GUV without PI does not show the activity [19]. Products of the F-BAR protein assembly have been studied in detail: a filament of F-BAR proteins linked end-to-end each other, and aligned to a coil on the tubular lipid bilayer [52,53]. However, the results in ref [19] imply that the assembly processes vary depending on the F-BAR protein species. In situ AFM observation in Figure 5 revealed the initial processes of the assembly of FBP17 molecules in prior to the tubular growth: adsorption, diffusion, and nucleation on the lipid bilayer membrane. The PI-derived microdomains provide the reaction site where the assembly starts, and the fluid region of the PI+PC membrane transfer reactant protein molecules to the reaction site. The result clearly demonstrated that the lipid microregions play roles depending on their composition and physical properties during the protein reaction on a lipid bilayer. 

## 4. Conclusions

Two regions with different physical properties existed in the PI+PC lipid bilayer: the submicron PI-derived domains that were relatively rigid and worked as diffusion obstacles, and the fluid surrounding region in which lipids freely diffused. An in situ observation of the FBP17 assembly in the PI+PC-SLB revealed that the PI-derived domain was the scaffold assisting the FBP17 adsorption for nucleation at the initial process. The fluid surrounding region provided FBP17 to the domain and grew the FBP17 assembly through the lateral diffusion. This study demonstrates the roles of the lipid domains during the protein reaction on the lipid membrane according as their compositions and physical properties. 

## Data Availability

Not applicable.

## Supplementary Materials

The following are available online at https://www.mdpi.com/article/10.3390/membranes11050339/s1; Supporting Information (Figure S1: Schematic of the diagonal illumination set-up for SPT; Figure S2: SPT results of PC-SLB without PI; and additional description about the analysis of AFM topography including Figure S3); Video S1 and S2: Movies of SPT of BODIPY-HPC and DiI-C18, respectively; Video S3: Sequential images of AFM topography obtained after addition of FBP17 to PI+PC-SLB.
