# Supplementary material for: Physical Properties and Reactivity of Microdomains in Phosphatidylinositol-Containing Supported Lipid Bilayer
Source: Membranes (Basel). 2021 May 3;11(5):339. doi: 10.3390/membranes11050339 (PMC8147626; doi:10.3390/membranes11050339)
Supplement: Supplementary file 1 [file membranes-11-00339-s001.zip › SupportingInformation_revised_210430.pdf]

Supporting information

Physical Properties and Reactivity of Microdomains in Phosphatidylinositol-Containing Supported Lipid Bilayer

Toshinori Motegi [1,*,¶], Kingo Takiguchi [2], Yohko Tanaka-Takiguchi [2], Toshiki Itoh [3] and Ryugo Tero [1,4,*]

1. Electronics-Inspired Interdisciplinary Research Institute, Toyohashi University of Technology, Toyohashi, 441-8580, Japan
2. Division of Biological Science, Graduate School of Science, Nagoya University, Nagoya, 464-8602, Japan
3. Biosignal Research Center, Kobe University, Kobe, 657-8501, Japan
4. Department of Applied Chemistry and Life Science, Toyohashi University of Technology, Toyohashi, 441-8580, Japan

¶ Present Address: Takasaki Advanced Radiation Research Institute, Quantum Beam Science Research Directorate, National Institutes for Quantum and Radiological Science and Technology (QST), 1233 Watanuki, Takasaki, Gunma 370-1292, Japan

* Correspondence: motegi.toshinori@qst.go.jp (T.M.); tero@tut.jp (R.T.)

Diagonal illumination setup for single particle tracking

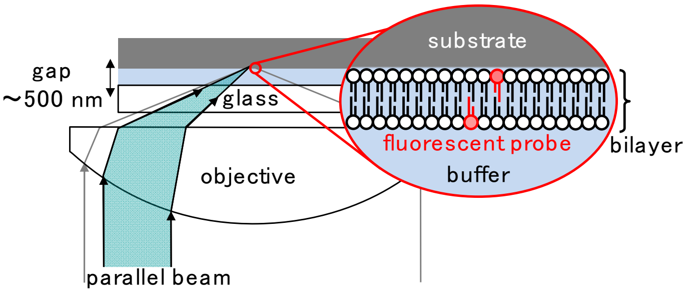

Figure S1. Schematic of the diagonal illumination setup.

Single particle tracking results of PC-SLB without PI

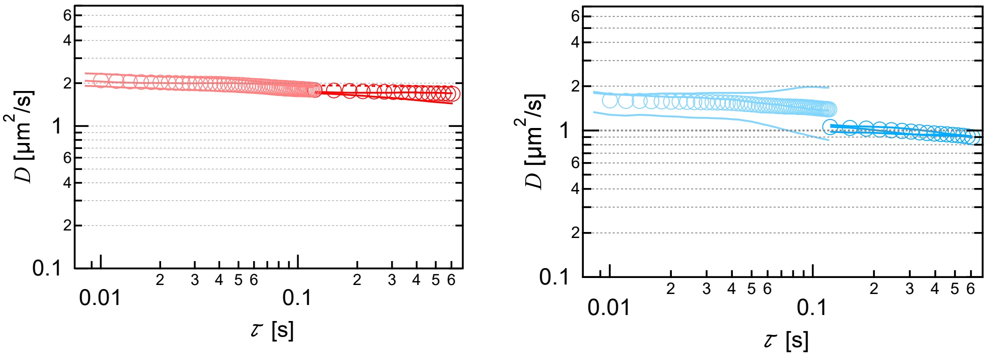

Figure S2. The dependence of D on τ plots obtained from the analyses of the average MSD-τ plots of DiI-C18 (left) and BODIPY-HPC (right) in PC-SLB without PI.

Analysis of AFM topography

The area of each depression domain was obtained from the AFM topographies with ImageJ, to calculate the average domain area (A_{domain}) and the area fraction of the domain. The domain was detected according to the height threshold from the images without any filtering processes. We estimated the size of the compartment, which is the domain-free region for the microscopic lipid diffusion, as follows. We assume the circular domains with A_{domain} are allocated periodically (Figure S3). The area of the unit cell (A) including a single domain is expressed as $A = A_{\mathrm{domain}} + A_{\mathrm{surr}}$, where A_{surr} is the area of the surrounding region around the domain. A_{surr} is calculated using the values of A_{domain} (1.69×10^4 nm^2) and the area fraction of the domain ($A_{\mathrm{domain}}/A = 0.24$) that were obtained from the AFM topographies. We assume A_{surr} as the area of the domain-free compartment and regard its size as the approximated diameter $2R = 2\sqrt{A_{\mathrm{surr}}/\pi} = 261$ (nm).

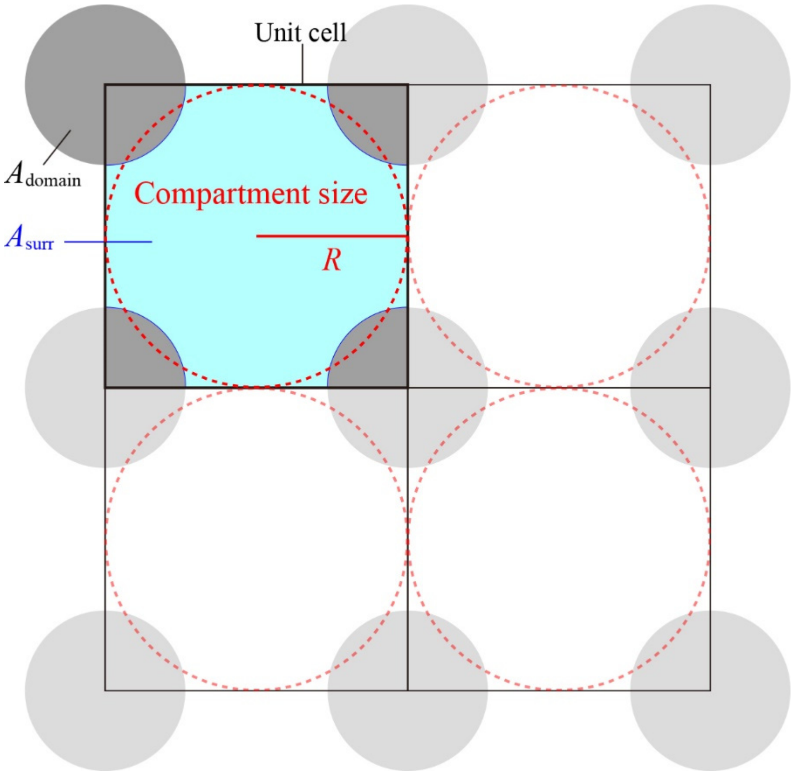

Figure S3. Schematic for estimating the domain-free compartment size.
